# Paying attention

**DOI:** 10.7554/eLife.99560

**Published:** 2024-06-10

**Authors:** Christian H Poth

**Affiliations:** 1 Neuro-Cognitive Psychology Group, Bielefeld University Bielefeld Germany

**Keywords:** vision, attention, saccades, eye movement, stimulus, Human

## Abstract

Intentional eye movements depend on where peripheral attention is voluntarily deployed beforehand, but they can be directed elsewhere shortly afterwards.

**Related research article** Goldstein AT, Stanford TR, Salinas E. 2024. Coupling of saccade plans to endogenous attention during urgent choices. *eLife* **13**:RP97883. doi: 10.7554/eLife.97883.

In many ways, we discover the world by looking at it. Through rapid eye movements – known as saccades – we can turn our gaze to specific objects, enabling us to pay attention to and scrutinize objects that are of high importance at the time. As well as this overt form of attention, we can also attend to objects in our periphery covertly (without moving the gaze).

Both covert and overt attention can be directed endogenously (on purpose) or exogenously – automatically attracted by a conspicuous stimulus, such as a sudden light (e.g., Carrasco, 2011). It has been shown that the brain processes required for covert attention and overt saccades are linked. Just before a saccade is directed at a target object, covert attention is already present, improving perception of the target (for example, see Deubel and Schneider, 1996; Hoffman and Subramaniam, 1995). Therefore, before a saccade begins, covert and overt attention seem to be coupled.

Most research on this topic has focused on how the coupling of covert attention and saccade planning supports visual perception. Now, in eLife, Allison Goldstein, Terrence Stanford and Emilio Salinas report insights into how paying covert attention to a peripheral object influences the subsequent saccade towards or away from the object (Goldstein et al., 2024).

The team (who are based at Wake Forest School of Medicine) asked participants to look at a central stimulus, which disappeared and was then replaced by two stimuli to the left and right. One of these peripheral stimuli (called the ‘cue’ stimulus) took one of two colors, indicating whether participants should look towards it (pro-saccade) or away from it (anti-saccade). The second peripheral stimulus, known as the non-cue, was always the same color. The location of the cue stimulus remained the same throughout a block of trials, meaning that participants knew its location and could attend to it covertly.

To investigate how this covert attendance of the cue stimulus affected the next saccade to the same or opposite location, Goldstein et al. combined the task with time pressure. Participants were instructed to saccade within a certain time after the initial center stimulus disappeared. The time between the center stimulus disappearing and the cue stimulus appearing varied. Sometimes, the cue followed shortly, informing the saccade. However, other times, the cue followed later, increasing the time pressure such that participants needed to saccade before the cue in order to meet the deadline. This was used to examine how performance (i.e., whether participants’ saccades were directed at the correct stimulus) was affected by these differences in cue time (before saccade).

The experiments showed that when the cue stimulus was visible too briefly to inform the saccade, performance was the same as it would if participants had guessed where to look (Figure 1B). When the cue was visible for longer, performance rose to a high level, indicating that the cue guided the saccade. Goldstein et al. also discovered that to saccade successfully, that is, to reach a set level of performance, participants needed the cue to be visible for about 30ms longer for anti-saccades than for pro-saccades (Figure 1B). This suggests that covert attention is directed at the cue initially, supporting saccades toward it, but this influence can be overcome quickly (in ~30ms), if the cue has indicated the saccade should be made away from the cue stimulus.

**Figure 1.**
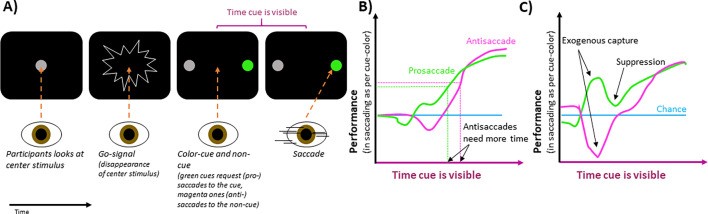
The effect of peripheral cues on intentional eye movements. (**A**) In an experimental trial, participants are asked to look at a central stimulus (gray circle in first screen). This stimulus then disappears, acting as a ‘go-signal’ (second screen). After this, two stimuli appear on the left and right of the center (third screen). One, known as the cue-stimulus is colored either green (as shown) or pink, while the other (the non-cue) remains gray. A green cue requests participants to look at it, whereas a pink one requests that they look away from it, towards the non-cue stimulus (fourth screen). The location of the cue was the same across a block of trials, ensuring participants knew where to attend. If cues quickly followed the go-signal, participants had enough time to make the requested saccades. If the cues were later, time pressure was high, and participants had to guess their saccade direction. (**B**) For pro-saccades (green), where participants are asked to look at the cue-stimulus, performance increases with the length of time the stimulus is present before the saccade. Anti-saccades (pink), where participants were asked to look away from the cue stimulus, require the cue to be visible for a longer time to reach this same level of performance (indicated by horizontal dashed lines), which is higher than that of chance (blue line). (**C**) When brighter cues are used, the gaze is captured more strongly, which increases or decreases performance, depending on whether participants were requested to saccade towards or away from the cue stimulus, respectively. After this initial exogenous capture, performance briefly moves in the other direction, suggesting capture suppression.

Image credit: Figure modified from Figures 1, 2 and 3 in Goldstein et al., 2024. Please note that the graphs in (**B**) and (**C**) are simplified depictions of the overall trends shown in Goldstein et al.’s figures and do not reflect accurate values.

Bright stimuli more strongly attract covert attention and exogenous overt saccades. By manipulating the brightness of cue and non-cue stimuli using dark backgrounds, Goldstein et al. influenced how saccades were automatically attracted to the stimuli, irrespective of whether the cue was pro- or anti-saccade. Brighter stimuli captured the gaze more strongly. Therefore, if participants were to look at a brighter cue stimulus, performance increased for a brief period (Figure 1C).

Interestingly, after this exogenous influence on performance, the relationship between performance and cue-viewing-time quickly moved in the opposite direction. This suggests that after the automatic capture of gaze, the exogenous influence seemed suppressed and facilitated saccades in the opposite direction. The brief suppression worked against the endogenous control of the saccade based on the cue, which manifested later as a gradual rise of performance toward its maximum. This suggests that the two forms of saccade control rely on independent underlying mechanisms.

In sum, Goldstein et al.’s findings reveal that saccades depend on where voluntary, covert attention is deployed before, but that this dependency can be overcome quickly. They also show that the gaze is attracted by conspicuous stimuli automatically, but that this attraction is suppressed afterwards, and eye movements become intentionally driven. The findings are based on a time pressure task that can require saccades to both possible locations to be planned before the cue, resulting in guesses if time pressure becomes too high. It is an open question how covert attention affects the next intentional saccade in more naturalistic situations, where upcoming saccades cannot be planned in such a way.
